# Case Report: Left atrial dissection after mitral valve replacement: intraoperative management under TEE guidance

**DOI:** 10.3389/fcvm.2024.1413713

**Published:** 2024-08-05

**Authors:** Mengyan Wang, Fucheng Ji, Jinfeng Zhou

**Affiliations:** Department of Anesthesiology, Qilu Hospital (Qingdao), Cheeloo College of Medicine, Shandong University, Qingdao, China

**Keywords:** atrial dissection, mitral valve replacement, left atrioventricular groove, TEE, surgical intervention

## Abstract

Left atrial dissection (LatD) is a very rare complication of cardiac surgery, but it is relatively more common in mitral valve surgery. Transesophageal echocardiography (TEE) plays an important role in timely detection of LatD and accurate assessment of the condition, which are key factors in determining the patient's prognosis. There are two different treatment options for patients with or without circulatory crisis caused by dissection hematoma, namely surgical management and conservative treatment. In this report, we used TEE to quickly detect the cause and severity of LatD, which assisted the surgeon in making appropriate surgical decisions. The patient was successfully surgically treated for LatD.

## 1 Introduction

Our patient was a 64-year-old woman who unexpectedly developed left atrial dissection (LatD) during mitral valve replacement surgery. Perioperative LatD is mostly iatrogenic. In this case, transesophageal echocardiography (TEE) was used to discover the site of injury that caused LatD, which is different from that in previously reported cases. Based on the evidence discovered by TEE, the surgeon was able to treat the patient successfully.

## 2 Case report

A 64-year-old woman was admitted to our cardiac surgery clinic with a complaint of chest tightness and dyspnea, which started 5 months ago without any apparent trigger. The symptoms worsened after exercise, accompanied by profuse sweating, dizziness, headache, abdominal bloating, and pain. She underwent TTE at a local hospital which revealed mitral valve prolapse (P2, P3 areas), severe mitral regurgitation, and left atrial enlargement. Because conservative treatment was ineffective, she was admitted to our hospital and referred for surgical intervention.

The laboratory test results were unremarkable. Coronary angiography showed no evidence of obstructive disease.

**Figure 1:**
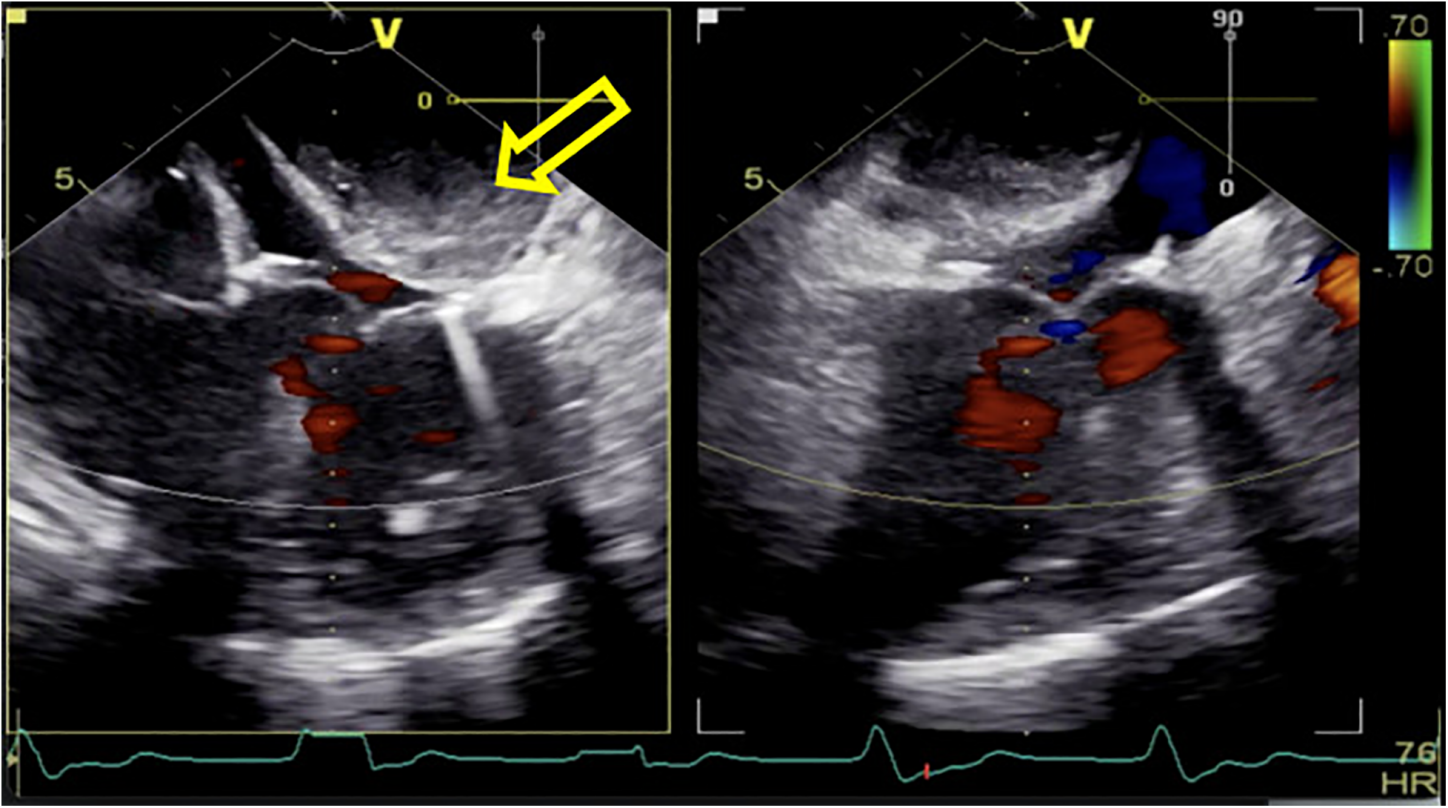
After being weaned from CPB, a rapidly increasing left atrial dissection hematoma (indicated by the yellow arrow) was visible in the posterior atrioventricular groove of the left atrial posterior wall. It gradually compressed the mitral valve annulus, causing relative stenosis of the left ventricular inflow tract.

Following routine cardiac general anesthetic procedures, TEE confirmed severe eccentric mitral regurgitation owing to prolapse of the middle scallop of the posterior mitral leaflet along with light tricuspid regurgitation and pulmonary valve regurgitation. The patient's left ventricular ejection fraction (LVEF) was normal (65%). A median sternotomy was performed, followed by standard right atrial and atrial septum access. Mitral valve replacement involved continuous suturing with 2-0 prolene thread, placement of a size 29 bioprosthetic valve, and ligation of the left atrial appendage with double 10-0 sutures. Subsequently, the aortic root was thoroughly deaired under TEE guidance, the aortic clamp was released, and the heart spontaneously started to re-beat. Unexpectedly, during the hemostasis process, the patient's blood pressure gradually decreased, requiring an increase in the dosage of adrenaline and noradrenaline to maintain an appropriate level. At this time, there was no obvious blood loss spots; we also excluded the possible use of medications such as protamine or antibiotics that could have triggered allergic reactions. To determine the cause of hypotension, we conducted an investigation using TEE and discovered a hematoma on the posterior wall of the left atrium, measuring approximately 3 × 4 cm in diameter (Figure 1). This hematoma was rapidly expanding and gradually compressing the mitral valve annulus.

**Figure 2:**
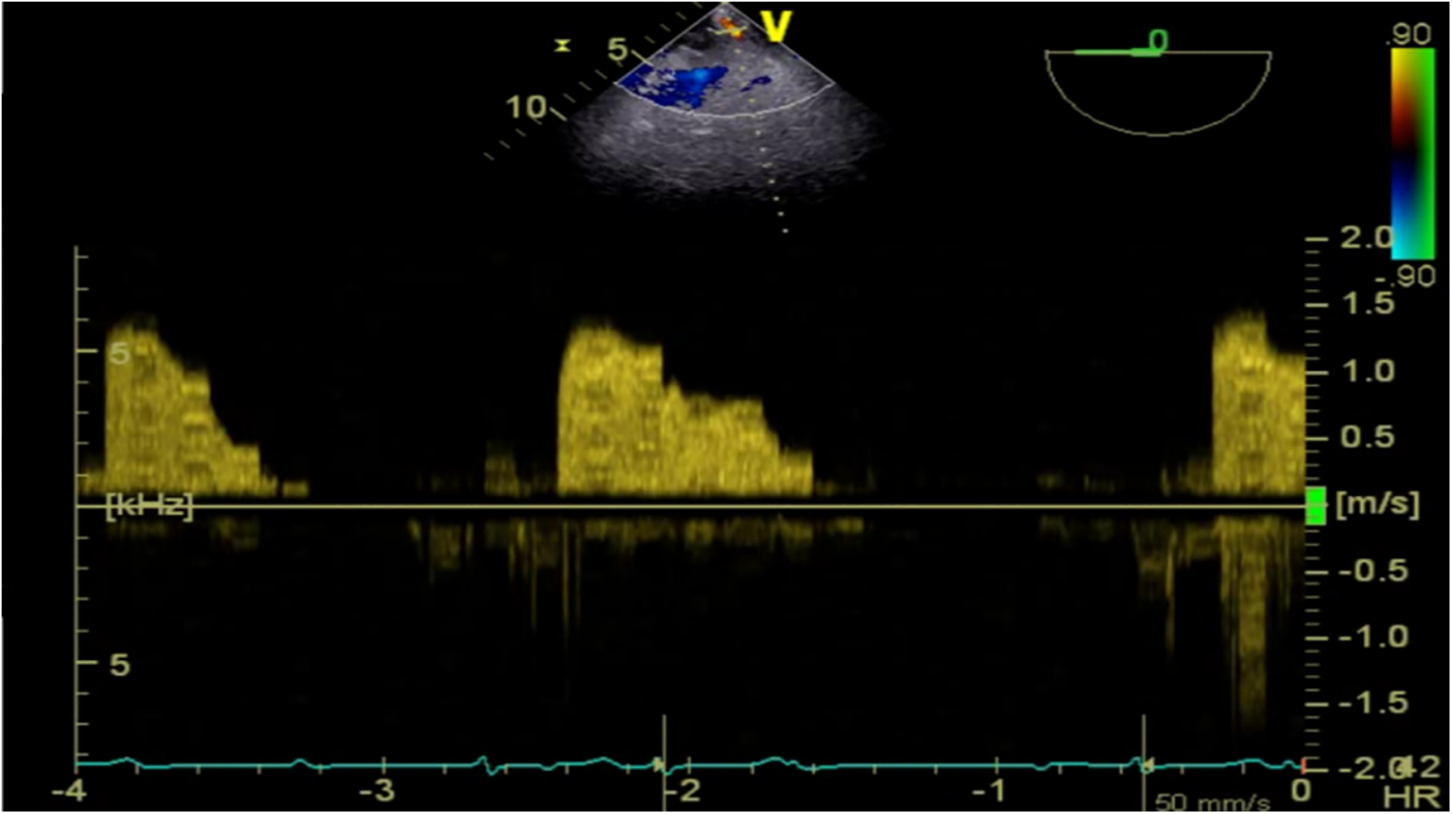
TEE detected pulsatile blood flow in the hematoma located at the left atrioventricular groove on the posterior wall of the left atrium.

**Figure 3:**
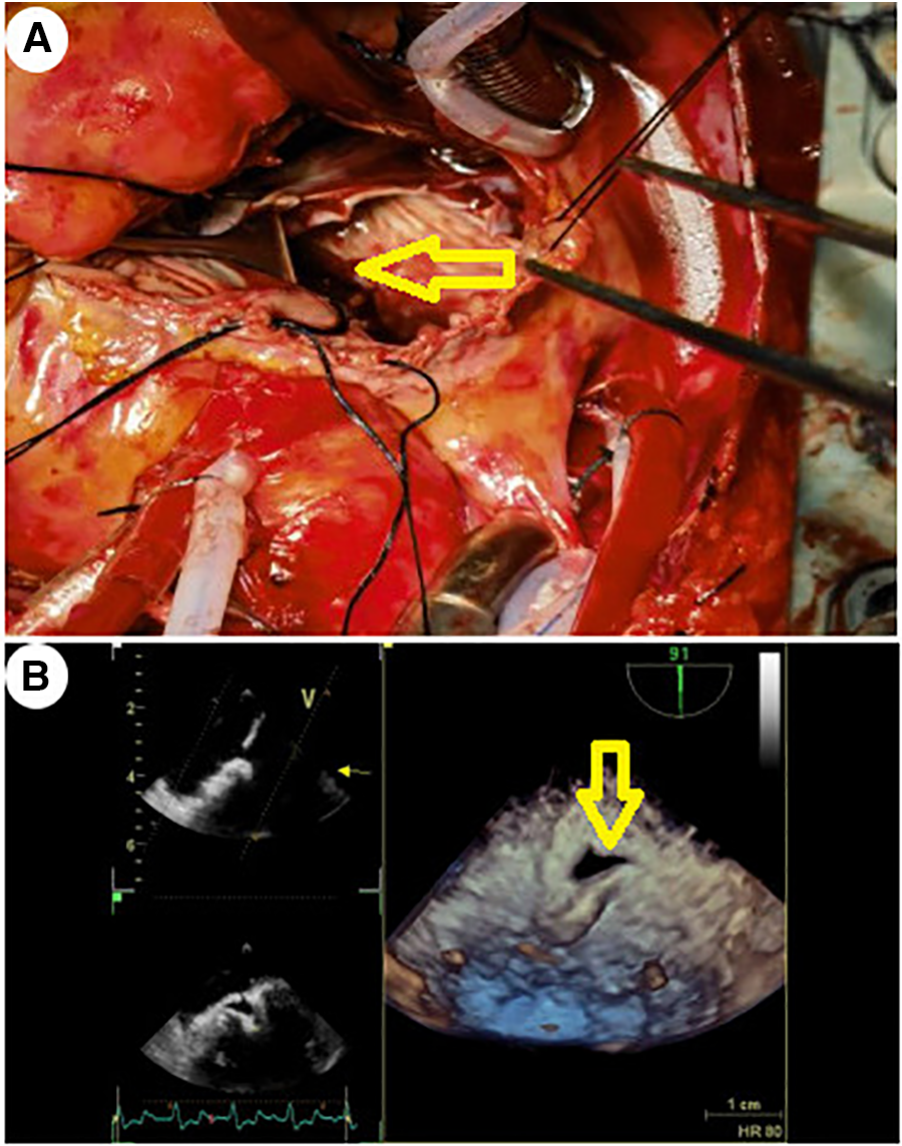
The yellow arrow indicates the site of incision and drainage. (**A**) Surgical treatment method: After confirming the integrity of the left atrial endocardium and the correct position of the artificial valve, the left atrial endocardium was incised for decompression and drainage. (**B**) TEE 3D reconstruction detected the drainage port of the left atrial endocardium incision for decompression.

**Figure 4:**
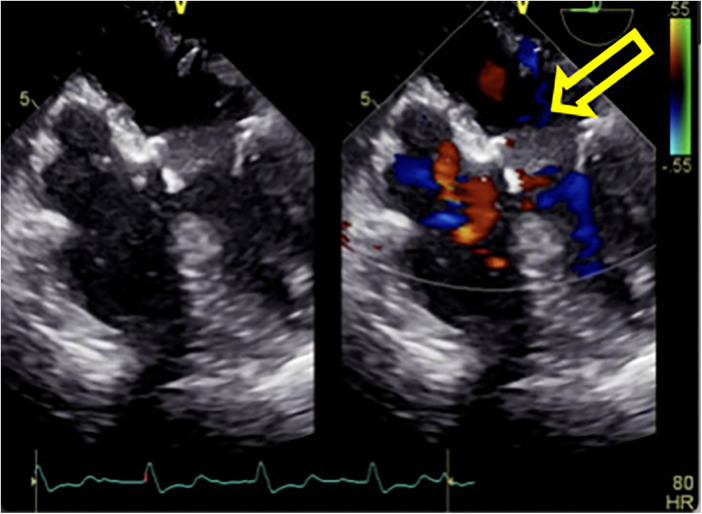
The hematoma on the posterior wall of the left atrium significantly decreased in size after incision of the left atrial endocardium for decompression and drainage.

Given this emergent situation, cardiopulmonary bypass was reinitiated to resolve the circulation crisis. The results of TEE monitoring showed that the left atrial dissection hematoma was compressing the left ventricular inflow tract, which accelerated blood flow through the mitral valve, causing mitral valve relative stenosis. In addition, TEE found pulsatile blood flow in the hematoma at the left atrioventricular groove on the posterior wall of the left atrium after the second cardiopulmonary bypass (Figure 2), suggesting that this blood flow was related to the left coronary circumflex branch. However, the relationship between the left circumflex branch and the dissection could not be determined. Because the criminal's blood flow (blood vessels) was located in the posterior atrioventricular groove and could not be explored through a surgical incision, we ultimately decided to incise the left atrial endocardium to drain the decompression hematoma through the left atrium (Figures 3, 4). The specific method applied was to reopen the right atrium and atrial septum along the original surgical incision. After confirming the correct position of the artificial valve and the integrity of the left atrial endocardium, the latter was incised for drainage and decompression, and the atrial septum and right atrium were resutured. The patient was admitted to the ICU for further management and discharged home after 16 days.

Follow-up transthoracic ultrasound at 15 and 25 days after surgery both showed heterogeneous echogenicity on the left atrial posterior wall, measuring approximately 26 mm × 12 mm, with no significant change compared to before. The ejection fraction remained at 63%.

## 3 Discussion

Left atrial dissection is defined as a false, blood-filled cavity or lumen from the mitral annular area to the left atrial free wall or interatrial septum that creates a new chamber with or without communications into the true left atrium (1). LatD is a rare complication of cardiac surgery, with reported incidence rates ranging from 0.16% to 0.84%, which is traditionally associated with mitral valve surgery. However, with the development of percutaneous coronary intervention (PCI) in recent years, the number of cases reported after radiofrequency ablation and PCI has also slightly increased (2). The reasons for LatD caused by different surgeries are different, such as the atrioventricular groove damage (3) during mitral valve surgery, atrial wall injury from ablation procedures (4), and coronary artery perforation during PCI (5). Genoni et al. (3) claimed that the most likely source of LatD hematoma during mitral valve surgery was left ventricular arterial blood. Intense traction of sutures in weak tissue can cause bleeding from the atrioventricular groove, which is not directed outward toward the pericardial cavity, rather toward the atrium itself, leading to spread of tissue and formation of a cavity.

In this report, we discuss a case of LatD that occurred after mitral valve surgery, but it had a unique presentation compared to previous reports. In previous reports, most cases of atrial dissection after mitral valve surgery were caused by surgical operation leading to damage to the left atrial endometrium. In this case, the LatD occurred during the valve annulus suturing process, especially when the suture needle in the posterior annulus was too deep, which damaged the coronary branches in the atrioventricular groove.

The overall mortality rate for left atrial dissection is reportedly 13.8% (6), emphasizing the importance of timely detection and accurate diagnostic assessment. Continuous monitoring through TEE plays a crucial role in both diagnosis and management. Almost all reports of LatD in the past two decades have relied on TEE monitoring. Before the TEE era, LatD could only be detected through intraoperative general examination or occasionally, by autopsy (7, 8). Nowadays, TEE is the first-choice diagnostic method, and in some cases, the origin and extent of LatD are clearly demonstrated by TEE, with duplication of the left atrial wall and vast movement of the dissected wall (9). The clinical application of 3D TEE can accurately evaluate the anatomical factors causing hemodynamic instability in LatD, such as the degree of compression on the left atrium and pulmonary vein opening, obstruction of the left ventricular inflow tract, and determination of whether there is blood flow communicating the true and false lumens that can cause a decrease in cardiac output and heart failure. Using continuous TEE monitoring of the mass during protamine administration, the temporal relationship between reversal of heparin and the changing appearance of the mass from mostly fluid-filled hypoechoic mass to a hyperechoic mass as the blood coagulated could be clearly visualized (10). Reliable TEE evaluation can help surgical teams make the correct treatment decisions immediately, block further deterioration of patient circulation status, and avoid the risk of postoperative secondary thoracotomy or even death. For unexpected left atrial hematoma, treatment decisions are mainly based on the stability of the patient's hemodynamics. However, there are numerous uncertain factors in the progression of left atrial dissection. Even for patients who choose conservative treatment for current stable hemodynamics, close monitoring should be carried out, especially continuous TEE monitoring (11).

In unstable patients, surgical treatment is the top priority. The purpose of surgical treatment is to eliminate hematoma, relieve compression, close the false lumen, and close the entrance (12). There are two methods for surgical treatment, one is internal drainage (3, 13, 14) and the other is entry closure (15–17) (i.e., by suturing the false cavity or tear layer). However, not all dissected hematomas' origins can be accurately located. Therefore, internal drainage often becomes the first choice for quickly alleviating hemodynamic collapse. In Genoni et al.'s (3) technique, internal drainage was applied to ensure drainage of a dissected left atrial wall into the right atrium. Hereby, the cavity of the left atrium is restored, while preventing systemic embolization and rupture by further increase of intracavitary pressure. Matteucci et al. (14) reported a new internal drainage method called atrial fenestration. In this case, during the extracorporeal circulation, owing to low blood pressure and laminar flow, a small-volume hematoma starts begins to develop. Additionally, because of the insufficient filling of the heart and the less standardized ultrasound section, any abnormalities that had already occurred can be easily overlooked.

Following the extracorporeal circulation stop, as the blood pressure increases, laminar flow shifts to pulsatile flow. Accelerated blood flow into the dissected layer rapidly enlarges the hematoma on the left atrium's posterior wall. This significantly obstructs the left ventricular inflow tract, with Doppler indicating a narrowed mitral valve orifice area of 1.5 cm^2^, equivalent to moderate mitral stenosis. Subsequently, the team opted for surgical intervention to counter the LatD's severe impact on circulation. They reestablished extracorporeal circulation and alleviated pressure by incising and draining the tense area on the left atrium's endocardial posterior wall. This surgical approach effectively minimized cardiac damage and shortened the extracorporeal circulation time for the patient.

## 4 Conclusion

TEE monitoring assists anesthesiologists gain a deeper understanding of the structural and functional aspects of the heart during surgery. It serves to facilitate efficient communication between anesthesiologists and surgeons. TEE monitoring serves as a crucial foundation for surgical decision-making. It allows for real-time, comprehensive assessment of the structure and function of the heart, enabling timely diagnosis of the causes of hemodynamic instability. In this case, reliable TEE monitoring facilitated the timely identification of the circulatory instability trigger, enabling the surgical team to make informed decisions and avoid unplanned reoperations. The case underscores the significance of incorporating TEE throughout the cardiac surgery process, emphasizing its role in providing timely and accurate monitoring information.

## Data Availability

The original contributions presented in the study are included in the article/Supplementary Material, further inquiries can be directed to the corresponding author.
